# Stranger Danger: Analyzing Offender Behaviors Based on Victim Approach Tactics in Sexual Homicide

**DOI:** 10.1002/bsl.2708

**Published:** 2024-12-06

**Authors:** Zena Rossouw, Eric Beauregard, Julien Chopin

**Affiliations:** ^1^ School of Criminal Justice and Criminology Texas State University San Marcos Texas USA; ^2^ School of Criminology Simon Fraser University Burnaby Canada; ^3^ School of Criminal Justice University of Lausanne Lausanne Switzerland

**Keywords:** crime script, manipulative approaches, rational choice, sexual homicide, stranger

## Abstract

Victims of sexual homicide may be deceived by perpetrators who use a friendly approach to gain access to them, making it difficult for the victim to assess the danger posed by the stranger. When investigating sexual homicides committed by strangers, investigators often lack direct information, including how the perpetrator gained access to the victim. To identify potential predictors of the approach method used in sexual homicides, this study analyzed the preferences and behaviors of sexual murderers who target strangers based on their approach method. The results of the logistic regression analysis showed that in comparison to offenders using “blitz” or “surprise” attacks, those using a deceptive “con” approach tended to have more male victims, exploit vulnerability, and exhibit post‐crime organization by relocating the victim's body and successfully disposing of the weapon used in the crime. Their crimes also more frequently involved oral sex and had lower rates of victim beating. This study discusses the investigative implications of these findings.

## 1 Introduction

The infamous American serial killer from the 1970s, Ted Bundy, has captivated the attention of numerous academic fields (James 2019; McClellan 2006; Williams 2020). Bundy was notorious for his ability to charm and manipulate his victims to gain access to them using a “con” or “ruse.” He would seek out a target and approach them by pretending to be an authority figure or someone needing assistance with a task. If his first plan failed, he would change his tactics, devising new strategies such as disabling a car to leave the victim stranded and vulnerable, but grateful for the help of a stranger (Michaud and Aynesworth 2000, 73; Williams 2020). Bundy's victim approach aligns with existing research on tactics used by those who commit sexual murder involving strangers (e.g., Dietz, Hazelwood, and Warren 1990; Gerard, Mormont, and Kocsis 2007; Keppel and Walter 1999; Stefanska et al. 2015). However, the problem is that this approach may not be known until a suspect has been identified or the crime is solved.

Similarly, between 2010 and 2017, eight men associated with Toronto's Gay Village disappeared before Bruce McArthur was charged with their murders. Despite McArthur's prior involvement in violent incidents, it may have been difficult to identify him as a potential murder suspect through database searches for three reasons: (1) he was not always charged for these incidents, (2) his prior assault was not classified as sexual, (3) and the amount of time that had passed since his last conviction (Brockbank 2018). In a particular incident, McArthur attempted to strangle a victim who managed to escape. McArthur self‐reported the incident to the police, claiming that he thought the victim was enjoying rough sexual activity and only realized something was wrong when the victim ran away (Brockbank 2018). The police found his statement plausible, leading to no arrest or charge. McArthur's strategic move makes it challenging to determine whether he intended to commit murder or assault, as the consensual nature of the initial interaction served to obscure his true intentions.

Understanding the offender's method of accessing a victim is important for two reasons. First, the approach phase is key in sexual homicides because, without access to a victim, the crime cannot occur (Beauregard, Rossmo, and Proulx 2007; Proulx and Beauregard 2009; Rossmo 2000). Second, knowing how someone became a victim of sexual murder can help piece together the story and identify where police might find evidence or clues to solve the crime (Rossmo 2021). Despite the significance of the perpetrator's victim approach strategy, little is known about it (Carter and Hollin 2010). This study aims to identify how law enforcement can determine the approach method used in cases of stranger‐perpetrated sexual homicide by analyzing offender behaviors and victim characteristics to distinguish between cons (tricks) and forceful tactics, such as blitz and surprise attacks (i.e., abduction or immediate assault).

### 1.1 Common Tactics for Approaching a Victim

In a study by Beauregard, Proulx, and St‐Yves (2007), it was found that almost half of the serial sexual offenders (48%) favored a “con approach” over quick attacks. The study participants explained that using a ruse allowed them to gain access to their victims with minimal violence or distractions. However, it is important to note that choosing one approach over another does not guarantee success in accessing the victim, as approximately 28% of those interviewed needed to change their focus from their original target to another victim.

Similarly, in a later study by Beauregard and Martineau (2013), it was found that between 1948 and 2010, two out of five sexual homicide offenders in Canada used a ruse to connect with their victims. In other words, nearly half of murderers were able to approach their victims under false pretenses (Beauregard and Martineau 2013, 1740). While not all victims are deceived by the trick, it gives offenders the ability to adapt their approach, unlike those who reveal their intentions by immediately attacking or attempting abduction (see Beauregard, Proulx, and St‐Yves 2007; Douglas et al. 1992).

People have individual predispositions, preferences, and circumstances ultimately influencing their decision on how best to approach a victim (Beauregard, Rossmo, and Proulx 2007; Rossmo 2000). They assess risks and benefits in varying ways, resulting in different methods for accomplishing their goals (Beauregard, Proulx, Rossmo, et al. 2007; Rossmo 2000). For example, in a study by MacCulloch et al. (1983), a participant who preferred to quickly attack a selected victim rather than using a ruse stated, “I didn't want to put myself on show in case I made a mess of it” (p. 23). Additionally, the environment in which they operate also plays a role in shaping their decision‐making process. Offenders who look for quick opportunities may be waiting for what Chan, Beauregard, and Myers (2015) describe as a “golden opportunity” (p. 239). This refers to situations with minimal or no surveillance, such as a lack of police presence, witnesses, or security cameras.

On the other hand, a sudden attack may not satisfy individuals such as Bundy, who take pleasure in exerting psychological control over their victims, or those who target victims more likely to resist (Meloy 2000; Michaud and Aynesworth 2000). The use of a con approach is significantly linked to the lifestyle and activities of the victim, indicating that external factors may necessitate disarming the victim, perhaps by establishing a friendly relationship, to maintain control of the situation (Beauregard, Proulx, and Rossmo 2007; Quinet 2011). For example, women in the sex trade frequently face violent clients and may resist if they sense danger. A study by Romero‐Daza, Weeks, and Singer (2010) highlighted the risks among women engaged in street‐level sex work. Their findings revealed that 60% of the women surveyed had experienced rape while working, and one in four reported surviving an attempted murder by a client.

### 1.2 Deciding How to Proceed: Weighing Risks and Unexpected Outcomes

The process of deciding how best to approach the victim is at the core of the rational choice theory. This theory proposes that individuals who commit crimes make decisions based on self‐interest, by analyzing the risks, efforts, and rewards (Cornish 1993; Cornish and Clarke 2008, 2). Just like everyone else, some offenders are better at weighing the pros and cons of their actions than others (Cornish and Clarke 2008, 2). Unexpected events, such as a victim's response or strength, can significantly change a perpetrator's behavior and the outcome of the crime (Cornish 1993; Proulx and Beauregard 2009). Chopin and Beauregard (2019), for example, found that in cases of rape where the victim resisted had the highest risk of escalating to sexual homicide, despite that the offender may not have initially intended to murder the victim. The homicide can become instrumental to the initial assault (Chopin and Beauregard 2019).

### 1.3 Crime Scripts: The Process of the Crime

Based on the available information and evidence, law enforcement often has to make educated guesses about what may have occurred during a crime. Therefore, understanding how crimes unfold, especially in complex cases like sexual violence, is necessary for efficiency and case solvency (Beauregard, Rossmo, and Proulx 2007). Researchers have identified a patterned process called a “crime script” which applies to cases of sexual violence, involving preparation, victim selection, the assault itself, and post‐crime behaviors (Beauregard, Proulx, Rossmo, et al. 2007; Cornish 1994; Piquero and Tibbets 2002; Proulx and Beauregard 2009; Rossmo 2000). A manipulative script, for example, involves a perpetrator creating opportunities to locate and interact with victims, such as engaging in the sex trade, leveraging their status to appear less threatening, and using tactics that do not stir suspicion from the victim and witnesses (Beauregard, Proulx, Rossmo, et al. 2007; Proulx and Beauregard 2009; Rossmo 2000). Understanding these patterns allows researchers and investigators to cluster similar behaviors to better understand how a crime may have unfolded.

The decision to commit a sexual assault can vary from impulsive to premeditated (Proulx and Beauregard 2009; Rossmo 2000). For example, a burglar breaking into an occupied home might decide to sexually assault the victim after a quick evaluation of the risks and benefits (Proulx and Beauregard 2009, 183; Rossmo 2000). The decision to murder the victim might be made to eliminate the witness to the crime (Proulx, Blais, and Beauregard 2007). On the other hand, some individuals may plan to commit the crime by considering where and how they will find a victim to fulfill their goals (see Rossmo 2000).

According to the rational choice theory, perpetrators may choose victims based on their physical features (as a high reward), vulnerability (as a low risk), and accessibility (as low effort) (Beauregard, Proulx, Rossmo, et al. 2007; Cornish and Clarke 1986, 2008; Keppel and Walter 1999; Martineau and Beauregard 2016; Meloy 2000; Proulx and Beauregard 2009; Quinet 2011). Anticipating obstacles and the victim's reaction, especially in cases involving strangers, can be challenging for the offender, as they are unfamiliar with the target and the situation (Beauregard, Rossmo, and Proulx 2007; Martineau and Beauregard 2016; Proulx and Beauregard 2009; Quinet 2011). Based on the victim and context, different approaches may be perceived as more effective in achieving the offender's objectives in one situation over another (Beauregard, Rossmo, and Proulx 2007; Rossmo 2000).

Deciding to impulsively commit a crime may lead the offender to act quickly, such as abducting a victim or attacking them opportunistically, similar to the scenario of the burglar mentioned earlier (Proulx and Beauregard 2009, 183). Others may have created the opportunity, such as when John Wayne Gacy targeted young vulnerable males he met in public places, including bus stations (James 2019). For Gacy, the con approach may have been the least risky option due to the potential risks posed by witnesses at the bus depot and the male victims, who could have fought back (Beauregard and Proulx 2007; Chopin and Beauregard 2021). Despite being strangers to him, they were willing to go with Gacy. He explained, “Everybody that ever came to my house, there was never a struggle, and nobody was ever forced into my house” (Rogers 2021). However, approaching strangers can be intimidating, which may cause some individuals to resort to aggressive tactics such as abduction or immediate attack, due to fear of rejection, embarrassment, or performance anxiety (Beauregard and Proulx 2002; MacCulloch et al. 1983; Stefanska et al. 2015).

The decisions made by offenders are clues about them. These choices can be grouped into specific types, referred to as typologies, which can help investigators understand the kind of offender they are dealing with. Arguably, the most referenced typologies are the “organized” and “disorganized” offenders, defined by the Federal Bureau of Investigation (FBI) based on police records and interviews with convicted sexual murderers (Burgess et al. 1986; Ressler, Burgess, and Douglas 1988).

Organized offenders are characterized by careful planning, the use of ruses to approach strangers, and controlled and manipulative behavior (Meloy 2000; Ressler et al., 1986; Ressler, Beauregard, and Martineau 1988). Examples of their strategic behavior includes the staging of the crime scene and moving the victim's remains (Meloy 2000; Ressler et al., 1986; Ressler, Beauregard, and Martineau 1988). Whereas “disorganized” offenders are impulsive, victimize acquaintances, and are likely to leave victims' remains at the crime scene (Meloy 2000; Ressler et al., 1986; Ressler, Beauregard, and Martineau 1988).

Since the FBI's work, researchers have continued to improve the accuracy of the typologies, leading to numerous classifications that can be difficult to apply in practice (Higgs et al. 2017). Efforts to consolidate the literature have resulted in three overarching subtypes: sadistic murderers, anger‐prone murderers, and witness elimination murderers (Beauregard and Proulx 2002, 2007; Chai, McCuish, and Beauregard 2021; Higgs et al. 2017; Proulx and Beauregard 2009; Miller 2014).

Offenders who act out of anger typically do not plan their actions (Beauregard and Proulx 2002; Chai, McCuish, and Beauregard 2021; Stefanska et al. 2015). Instead, the urge to commit a sexual offense usually arises before they select a victim; often someone they know (Higgs et al. 2017, 6). This can lead to situations that start consensually but escalate into violence, catching the victim off guard (Beauregard, Rossmo, and Proulx 2007; Proulx and Beauregard 2009; Stefanska et al. 2015). Sometimes, they might approach victims casually or initially use deception to gain access to their location, such as their home, and then suddenly attack the victim (Keppel and Walter 1999).

While some victims comply initially, most end up resisting (Proulx and Beauregard 2009). The actions of the offender are driven by emotions such as anger, frustration, and humiliation (Beauregard and Proulx 2002; Proulx and Beauregard 2009), which may originate from long‐standing issues and resentment toward women (Beech, Fisher, and Ward 2005; Higgs et al. 2017). Offenders in this category often exhibit narcissistic and dependent personality disorders, leading to a lifestyle marked by promiscuity, antisocial behavior, and substance abuse (Beauregard and Proulx 2002; Proulx and Beauregard 2009; Stefanska et al. 2015). Although their crimes tend to be more physically violent, such as repeatedly stabbing the victim, some offenders may also subject victims to humiliation or foreign objects insertion (Beauregard and Proulx 2002; Higgs et al. 2017).

Once they have committed the crime, most will leave the body at the scene (Beauregard and Proulx 2002; Kocsis, Cooksey, and Irwin 2002). While they might have a history of hypersexuality, prior rape convictions are rare, though physical abuse toward women is more common (Proulx and Beauregard 2009; Stefanska et al. 2015). Interestingly, offenders fitting the “anger” model often surrender to authorities and take responsibility for their actions (Beauregard, Proulx, and St‐Yves 2007).

Sadistic murderers are primarily motivated by deviant sexual fantasies, where murder is an integral component of their crime (Beauregard and Proulx 2002; Dietz, Hazelwood, and Warren 1990; Stefanska et al. 2015). Their offenses entail thoughtful planning. They target strangers, engaging them under false pretenses, such as soliciting services from the sex trade or befriending the victim (Beauregard and Proulx 2002; Beauregard, Rossmo, and Proulx 2007; Dietz, Hazelwood, and Warren 1990; Meloy 2000).

Among the sexual acts forced upon victims, penetration, fellatio, and foreign object insertion are most common in this typology (Balemba, Beauregard, and Martineau 2014; Beauregard and Proulx 2002; Beech, Fisher, and Ward 2005; Dietz, Hazelwood, and Warren 1990; Gerard, Mormont, and Kocsis 2007). The victim may experience multiple sexual acts during the crime, rather than just one isolated incident. According to Dietz, Hazelwood, and Warren (1990), 67% of these offenders compel their victims to endure at least three sexual acts.

After the offense, most offenders will move the body (Beauregard and Proulx 2002). The heightened level of organization exhibited by these offenders may explain why they are associated with higher victim counts (Kocsis, Cooksey, and Irwin 2002). Interestingly, crimes committed by sadistic individuals are likely to be solved, but it may take the police longer to apprehend the perpetrator (Reale, Beauregard, and Martineau 2017).

Offenders classified as “opportunistic” or who kill as a means to eliminate witnesses are driven by practical or situational reasons. They may use a con approach to gain access to a victim for the purpose of sexually assaulting them (Stefanska et al. 2015). Although these offenders may not intend to kill, they will resort to violence to overcome resistance or avoid getting caught (Beech, Fisher, and Ward 2005; Higgs et al. 2017; Proulx and Beauregard 2009; Proulx, Blais, and Beauregard 2007; Stefanska et al. 2015). Proulx, Blais, and Beauregard (2007) labeled these offenders as “witness elimination” types.

Cases of rape that escalate to sexual murder are distinguished by their lack of behavioral consistency (Higgs et al. 2017). Unlike other forms of sexual murder, the violence in these cases is not necessarily associated with deviant sexual interests or triggered by negative emotions. As a result, the typical patterns observed in other types, such as sadistic behavior and extreme violence, are not consistent across these cases (Balemba, Beauregard, and Martineau 2014; Kocsis, Cooksey, and Irwin 2002; Stefanska et al. 2015). Instead, the process for carrying out the crime may vary, presenting evidence of mixed typologies (Proulx 2008).

### 1.4 The Current Study

In cases of sexual murders involving strangers, there is often limited context to discern what may have occurred. Traditional investigative methods, such as examining the victim's known contacts and associates, may not always generate new leads (Michaud and Aynesworth 2000, vii). Earlier research has shown that the offender's selected victim approach method can suggest how long the offender spent with the victim before the crime, their level of organization, sadism, and whether they may have been involved in additional sexually violent acts, all of which are helpful to guide the investigative efforts (Beauregard and Proulx 2002; Dietz, Hazelwood, and Warren 1990; Higgs et al. 2017; Proulx and Beauregard 2009). Despite its significance and association with certain offender behaviors, the approach method has not been the focus of research examining differences across crime phases and considering contextual factors. This study aims to fill that gap by comparing cases based on the offender's chosen approach to the victim.

Understanding how perpetrators gain access to stranger victims is helpful for identifying situations where witnesses may have unwittingly encountered the perpetrator. This is especially important in cases where initial interactions may appear consensual, giving the offender the opportunity to plausibly explain the situation if questioned by the police (similar to Bruce McArthur). In instances where the offender has no charges or convictions, this may result in the perpetrator being excluded from suspect lists or being viewed as a less likely suspect compared to those charged with a related crime, such as sexual assault (Beauregard and Martineau 2013; Greenall and Richardson 2015; Rossmo 2021).

As the investigation unfolds, crimes involving strangers continue to pose significant challenges, including sifting through a large volume of information, managing lengthy suspect lists, a lack of witnesses, and time constraints due to the risk of future incidents (Beauregard and Proulx 2002; Beauregard, Rossmo, and Proulx 2007; Dietz, Hazelwood, and Warren 1990; Greenall and Richardson 2015; Proulx and Beauregard 2009; Rossmo 2021). To assist investigators in prioritizing incoming information and focusing their efforts effectively, our study aimed to address the following research questions:

1. Do significant differences exist in the behaviors of sexual homicide offenders who use a manipulative approach (“con approach”) compared to those who employ alternative tactics (blitz or surprise) when targeting stranger victims?
2. Do contextual factors, such as victim characteristics or environmental conditions, significantly influence the offender's choice of approach method (manipulative, blitz, or surprise) in sexual homicide cases?

Based on current research on sexual murder, it is hypothesized that individuals who use a “con approach” may demonstrate more strategic behavior and share characteristics with sadistic offenders during their crimes. Those using alternative tactics may appear less planned and employ more violence. To test this hypothesis, single‐offender sexual homicide cases in Canada and France were studied, where the victim was a stranger and the offender used either a con, blitz, or surprise attack to initiate contact.

## 2 Method

### 2.1 Sample

The sample for this study was taken from the Sexual Homicide International Database (SHIeID), consisting of 762 cases of sexual homicide that occurred in France (*n* = 412) and Canada (*n* = 350) between 1948 and 2017. The data was input by analysts trained in coding violent crime (see Chopin and Beauregard 2021). Each case met the following criteria: (a) all cases were completed homicides (i.e., no attempted homicides), (b) each case featured at least two sexual elements (i.e., evidence of sexual activity or sexual motivation), and (c) involved a single offender. The sample was further refined to include only offenses where the victim was a stranger to the offender. The final sample included 323 cases (*N* = 323). A case was considered a stranger attack when both the offender and victim were unfamiliar at the time of the crime (Chopin and Beauregard 2021). A “sexual element” was identified using the FBI criteria, which includes (a) victim's attire or lack of attire; (b) exposure of victim's sexual parts; (c) sexual positioning of the victim's body; (d) insertion of foreign objects into victim's body cavities; (e) evidence of other sexual activity, interests, or sadistic fantasies (Chopin and Beauregard 2021, 2022; Ressler, Burgess, and Douglas 1988).

The study focused on stranger‐perpetrated sexual homicides for two reasons. Firstly, studies indicate that acquaintance sexual homicides exhibit different offending patterns when compared to those committed by strangers (Beauregard and Field 2008; Higgs et al. 2017). Variations in the relationship between the victims and perpetrators can influence the course of the crime, affecting factors such as target selection, victim control methods, victim age, level of violence, and body disposal methods. Secondly, offenses committed by strangers are amongst the most challenging cases to solve (Dietz, Hazelwood, and Warren 1990; Meloy 2000; Salfati and Canter 1999).

### 2.2 Measures

#### 2.2.1 Dependent Variable: Con Approach Versus Other (i.e., Blitz or Surprise Attacks)

To be classified as a con approach (coded as 1), the offense involved manipulation or trickery to gain access to the victim. Specifically, this approach involves deceiving the victim to achieve the perpetrator's goals, such as convincing the victim to enter their vehicle or feigning the need for assistance, which is typically non‐threatening (Beauregard, Proulx, Rossmo, et al. 2007). Other approach methods (coded as 0) encompass coercive tactics, such as a “blitz approach” involving a physical assault on the victim, surprising the victim through abduction, or threats (Beauregard, Proulx, Rossmo, et al. 2007; Beauregard, Rossmo, and Proulx 2007; Chopin and Beauregard 2019).

#### 2.2.2 Independent Variables

Based on previous studies, 24 variables related to an offender's behaviors and decisions throughout the crime process were included for analysis and were examined under three phases and sub‐categories: (a) pre‐crime: victim characteristics and routine activities, (b) crime: sexual acts, and violence, and (c) post‐crime phase: body and weapon disposal.

#### 2.2.3 Pre‐Crime

##### 2.2.3.1 Characteristics of the Victim

As presented in Table 1, the first subcategory includes four variables related to the victim's characteristics. Victim variables were selected because previous studies have shown that sexual murderers may target their victims, especially those who belong to a vulnerable population (e.g., Beauregard and Proulx 2007; Chopin & Beauregard 2021). All variables are dichotomous (0 = no, 1 = yes): (1) the victim was targeted, (2) the victim was older than 16, (3) the victim's sex (0 = male, 1 = female), (4) the victim led a vulnerable lifestyle (i.e., psychological disorders and drug or alcohol abuse), and (5) the victim was a loner.

**TABLE 1:** Descriptives and coding for the variables included in the analysis.

Variable	Coding	Frequency (*n* = 323)	%	Range
Pre‐crime phase = 1 (yes)				
Victim targeted	0 = no, 1 = yes	73	22.6	
Victim characteristics				
Victim > 16 years	0 = no, 1 = yes	268	83	
Victim sex	0 = male, 1 = female	284	87.9	
Victim vulnerable lifestyle	0 = no, 1 = yes	77	23.8	
Victim was a loner	0 = no, 1 = yes	20	6.2	
Routine activities				
Victim was engaged in domestic activity	0 = no, 1 = yes	80	24.8	
Victim was partying	0 = no, 1 = yes	24	7.4	
Victim engaged in risky activity	0 = no, 1 = yes	33	10.2	
Crime phase = 1 (yes)				
Sexual acts				
Penetration	0 = no, 1 = yes	196	60.7	
Fellatio	0 = no, 1 = yes	64	19.8	
Foreign object insertion	0 = no, 1 = yes	33	10.2	
Unusual acts	0 = no, 1 = yes	42	13	
Number of sexual acts	0 = no, 1 = yes	1.5^a^	1.22^b^	0–5
Violence				
Lethal weapon involved	0 = no, 1 = yes	137	42.4	
Other weapon involved	0 = no, 1 = yes	58	18	
Multiple acts of violence	0 = no, 1 = yes	238	73.7	
Offender beat the victim	0 = no, 1 = yes	140	43.3	
Strangulation/asphyxiation	0 = no, 1 = yes	146	45.2	
Post crime phase = 1 (yes)				
Weapon was recovered	0 = no, 1 = yes	54	16.7	
Case solved	0 = no, 1 = yes	315	97.5	
Body was recovered outdoors	0 = no, 1 = yes	138	42.7	
Body was discovered partly in water	0 = no, 1 = yes	19	5.9	
Body was buried	0 = no, 1 = yes	30	9.3	
Body was moved	0 = no, 1 = yes	83	25.7	

^a^ Represents the mean.

^b^ Represents the standard deviation.

##### 2.2.3.2 Routine Activities

For routine activities, the three variables related to the victim's activities before the crime were selected as researchers have found that offenders may be successful in carrying out their crimes due to learned strategies, which include selecting vulnerable victims and planning the approach carefully (Chai et al. 2022; Reale and Beauregard 2018). Certain activities, such as sex work, partying, and hitchhiking, have been found to increase the vulnerability of victims (Quinet 2011). Additionally, most non‐serial sexual murders occur in either the victim's home (Grubin 1994; Langevin et al. 1988; Roberts and Grossman 1993) or public places (Beauregard and Martineau 2013; Roberts and Grossman 1993). Therefore, the variables selected were: (6) the victim was engaged in a domestic activity, (7) the victim was partying, and (8) the victim was engaged in a risky activity (i.e., sex work or hitchhiking). All variables were coded dichotomously (0 = no; 1 = yes) (Table 1).

#### 2.2.4 Crime Phase

##### 2.2.4.1 Sexual and Violent Acts

The crime phase involves 10 variables reflecting behaviors related to sexual and physical violence, as researchers have found variations in violence and sexual acts against the victim (Beauregard and Proulx 2007; Chopin and Beauregard 2022). These behaviors, which indicate criminal expertise and deliberate decisions, can provide insight into the type of sexual offender. For example, sadistic offenders, who tend to be sexually motivated, may involve multiple sexual acts, including the use of foreign objects (Dietz, Hazelwood, and Warren 1990; Meloy 2000). Whereas “angry” and “grievance murderers,” motivated by resentment, typically engage in multiple acts of violence (Gerard, Mormont, and Kocsis 2007; Kocsis, Cooksey, and Irwin 2002; Stefanska et al. 2015). Research suggests that personal weapons, such as hands, used to strangle the victim, edged weapons like knives, and intimate violence, such as beatings, are frequently used in cases of sexual homicide (Chan & Heide 2009; Beauregard & Martineau 2013; Gerard, Mormont, and Kocsis 2007). Except for one continuous variable (the number of sexual acts; range = 0–5), all variables were coded dichotomously (Table 1). The sexual acts included the following: (9) Penetration, (10) fellatio, (11) foreign object insertion, (12) unusual acts, and (13) the number of sexual acts. Unusual acts are defined as “the presence of at least one of the following extreme crime scene behaviors: carving on the victim, evisceration, skinning, cannibalism, and vampirism” (Sun, Beauregard, and Chopin 2023, 185).

Lastly, under the violence category, the following were included in the analysis: (14) the use of a lethal weapon (i.e., gun or a knife), (15) the use of other weapons (i.e., blunt object or ligature), (16) multiple acts of violence, (17) whether the offender beat the victim, and (18) whether they use strangulation and/or asphyxiation.

#### 2.2.5 Post‐Crime

The selection of the post‐crime variables is rooted in existing research demonstrating that offender characteristics influence their post‐mortem management of the victim's body and efforts to delay police detection (Beauregard and Martineau 2014; Salfati and Canter 1999). The decisions are associated with typologies of sexual murderers, which are linked to a preferred victim approach style. As an example, Dietz, Hazelwood, and Warren (1990) found that sadistic offenders often approach victims using a con and tend to conceal the victim's body. Additionally, a dichotomous variable was included to indicate the case status, as research has shown how offender characteristics affect their post‐mortem handling of the victim's body and efforts to delay police detection (Beauregard and Martineau 2014; Salfati and Canter 1999). The variables included were: (19) whether the weapon was recovered, (20) whether the case was solved, (21) whether the body was recovered outdoors, (22) whether the body was discovered partly in water, (23) whether the body was buried, and (24) whether the body was moved.

### 2.3 Analytical Strategy

A two‐step analytical process was used to analyze the data. First, descriptive statistics were run to explore the extent to which behaviors and victim characteristics were evident in the crime commission process of sexual homicide involving stranger victims (Table 1). Next, a bivariate analysis (i.e., chi‐square and an independent *t*‐test for the continuous variable) was conducted to examine the relationships between the dependent variable (whether a con approach was used by the offender, 1 = yes, 0 = no) and the independent variables. Variables with *p*‐values less than 0.05 were retained to ensure all relevant variables were included in the logistic regression analysis (Hosmer and Lemeshow 2000). Lastly, to evaluate the performance of the binary classification model developed through logistic regression, a Receiver Operating Characteristic (ROC) curve was run.

## 3 Results

As shown in Table 2, offenders who targeted vulnerable victims, such as those with lifestyles characterized by substance abuse and psychological disorders (*χ* ^2^ (1) = 3.95, *p* = 0.047, *φ* = 0.11), and those engaged in risky activities like hitchhiking or the sex trade (*χ* ^2^ (1) = 6.34, *p* = 0.012, *φ* = 0.14), demonstrated a higher inclination toward employing a con approach. On the other hand, stranger‐perpetrated sexual homicides involving victims that were aged 16 or older (*χ* ^2^ (1) = 3.97, *p* = 0.046, *φ* = −0.11), female (*χ* ^2^ (1) = 13.02, *p* < 0.001, *φ* = −0.20), and involved in partying prior to the offense (*χ* ^2^ (1) = 4.97, *p* = 0.026, *φ* = −0.12) displayed significant associations with other approach methods, such as blitz or surprise attacks.

**TABLE 2:** Bivariate analysis of stranger sexual homicide: A comparison between con approach and other approaches (*N* = 323).

Variable	Other (*n* = 145)	Con approach (*n* = 178)	Total (*N* = 323)	*χ* ^2^/*t*, (df), Phi/*d*
	*M* (SD)/% (*n*)	*M* (SD)/% (*n*)		
Pre‐crime phase				
Victim targeted	22.1 (32)	23 (41)	22.6 (73)	*χ* ^2^ (1) = 0.01, *φ* = 0.01
Victim characteristics				
Victim > 16 years	87.6 (127)	79.2 (141)	83 (268)	*χ* ^2^ (1) = 3.97, *φ* = −0.11*
Victim sex	95.2 (138)	82 (146)	87.9 (284)	*χ* ^2^ (1) = 13.02, *φ* = −0.20***
Victim lifestyle vulnerable	18.6 (27)	28.1 (50)	23.8 (77)	*χ* ^2^ (1) = 3.95, *φ* = 0.11*
Victim was a loner	5.5 (8)	6.7 (12)	6.2 (20)	*χ*2 (1) = 0.21, *φ* = 0.03
Routine activities				
Victim was engaged in domestic activity	26.9 (39)	23 (41)	24.8 (80)	*χ* ^2^ (1) = 0.64, *φ* = −0.05
Victim was partying	11 (16)	4.5 (8)	7.4 (24)	*χ* ^2^ (1) = 4.97, *φ* = −0.12*
Victim engaged in risky activity	5.5 (8)	14 (25)	10.2 (33)	*χ* ^2^ (1) = 6.34, *φ* = −0.14*
Crime phase				
Sexual acts				
Penetration	57.2 (83)	63.5 (113)	60.7 (196)	*χ* ^2^ (1) = 1.31, *φ* = 0.06
Fellatio	13.1 (19)	25.3 (45)	19.8 (64)	*χ* ^2^ (1) = 7.46, *φ* = 0.15**
Foreign object insertion	12.4 (18)	8.4 (15)	10.2 (33)	*χ* ^2^ (1) = 1.39, *φ* = −0.07
Unusual acts	14.5 (21)	11.8 (21)	13 (42)	*χ* ^2^ (1) = 0.51, *φ* = −0.04
Number of sexual acts	1.35 (1.18)	1.61 (1.25)	1.50 (1.22)	*t* (321) = −1.94, *d* = 0.21
Violence				
Lethal weapon involved	45.5 (66)	39.9 (71)	42.4 (137)	*χ* ^2^ (1) = 1.04, *φ* = −0.06
Other weapon involved	13.1 (19)	21.9 (39)	18 (58)	*χ* ^2^ (1) = 4.21, *φ* = 0.11*
Multiple acts of violence	77.9 (113)	70.2 (125)	73.7 (238)	*χ* ^2^ (1) = 2.45, *φ* = −0.09
Offender beat the victim	49.7 (72)	38.2 (68)	43.3 (140)	*χ* ^2^ (1) = 4.27, *φ* = −0.12*
Strangulation/asphyxiation	43.4 (63)	46.6 (83)	45.2 (146)	*χ* ^2^ (1) = 0.326, *φ* = 0.03
Post crime phase				
Weapon was recovered	23.4 (34)	11.2 (20)	16.7 (54)	*χ* ^2^ (1) = 8.56, *φ* = −0.16**
Case solved	97.9 (142)	97.2 (173)	97.5 (315)	*χ* ^2^ (1) = 0.18, *φ* = −0.02
Body was recovered outdoors	40 (58)	44.9 (80)	42.7 (138)	*χ* ^2^ (1) = 0.80, *φ* = 0.05
Body was discovered partly in water	9 (13)	3.4 (6)	5.9 (19)	*χ* ^2^ (1) = 4.52, *φ* = −0.12*
Body was buried	9.7 (14)	9 (16)	9.3 (30)	*χ* ^2^ (1) = 0.04, *φ* = −0.01
Body was moved	19.3 (28)	30.9 (55)	25.7 (83)	*χ* ^2^ (1) = 5.62, *φ* = 0.13*

* *p* < 0.05; ** *p* < 0.01; *** *p* < 0.001.

In the crime phase, three variables were significantly associated with the offender's approach. Specifically, instances involving fellatio (*χ* ^2^ (1) = 7.46, *p* = 0.006, *φ* = 0.152) and the use of alternative weapons, such as ligatures and blunt objects (*χ* ^2^ (1) = 4.21, *p* = 0.040, *φ* = 0.11), were significantly related to stranger‐perpetrated sexual homicides where the con approach was used. Cases where the offender beat the victim (*χ* ^2^ (1) = 4.27, *p* = 0.039, *φ* = −0.12) were significantly associated with other approach methods such as “blitz” or surprise.

During the post‐crime phase, three variables were significantly associated with the offender's selected approach. Those who used coercive approaches were associated with cases where the victim's body was partially submerged in water (*χ* ^2^ (1) = 4.52, *p* = 0.034, *φ* = −0.12) and a weapon was recovered (*χ* ^2^ (1) = 8.56, *p* = 0.003, *φ* = −0.16). On the other hand, those who used a con approach were significantly more likely to move the victim's body (*χ* ^2^ (1) = 5.62, *p* = 0.018, *φ* = 0.13).

Based on the results from the bivariate analysis, 11 variables were retained for the logistic regression. As shown in Table 3, the model showed statistical significance (*χ* ^2^ (11) = 68.41, *p* < 0.001), yielding a Nagelkerke *R* ^2^ of 25.5% and accurately classifying 67.2% of cases. However, the Hosmer and Lemeshow test indicated a lack of adherence to the logistic distribution assumption (*p* = 0.005), likely influenced by two outlier cases. After excluding the outliers, the model adhered to the logistic distribution assumption (*p* = 0.371) (Table 3). Similarly, a revised logistic regression model (Model 2) was created, excluding the outliers (*n* = 321). Model 2 adhered to the logistic distribution assumption, as indicated by a non‐significant Hosmer Lemeshow test result (*p* = 0.371). The overall significance remained unchanged after excluding outliers (*χ* ^2^ (9) = 69.27, *p* < 0.001); however, Nagelkerke *R* ^2^ increased from 0.26 (Model 1) to 0.28 (Model 2). Predictive accuracy slightly improved from 67.2% (Model 1) to 67.6% (Model 2). All variables retained their significance in the same direction.

**TABLE 3:** Logistic regression of stranger sexual homicide: A comparison between con approach and other approaches (*N* = 323).

Variables	Including outliers				Excluding outliers			
	(*N* = 323)		95% CI		(*N* = 321)		95% CI	
	B (SE) OR	*p*	LL	UL	B (SE) OR	*p*	LL	UL
Pre‐crime phase = 1 (yes)								
Victim > 16 years	−0.57 (0.35) 0.56	0.103	0.28	1.12	−0.65 (0.36) 0.52	0.069	0.26	1.05
Victim sex	−1.69 (0.48) 0.18	< 0.001***	0.07	0.47	−1.74 (0.48) 0.18	< 0.001***	0.07	0.45
Victim lifestyle vulnerable	0.88 (0.32) 2.40	0.006**	1.29	4.49	0.92 (0.33) 2.52	0.005**	1.33	4.77
Victim was partying	−1.18 (0.51) 0.31	0.022*	0.11	0.84	−1.41 (0.54) 0.24	0.008**	0.09	0.70
Crime phase = 1 (yes)								
Fellatio	0.89 (0.33) 2.43	0.007**	1.27	4.65	0.92 (0.33) 2.5	0.006**	1.30	4.81
Victim engaged in risky activity	1.07 (0.47) 2.91	0.022*	1.17	7.23	1.04 (0.47) 2.82	0.027*	1.13	7.05
Other weapon involved	0.63 (0.36) 1.88	0.079	0.93	3.80	0.62 (0.36) 1.85	0.091	0.91	3.77
Offender beat the victim	−0.58 (0.26) 0.56	0.025*	0.34	0.93	−0.64 (0.26) 0.53	0.014*	0.32	0.88
Post crime phase = 1 (yes)								
Weapon was recovered	−0.82 (0.35) 0.44	0.023*	0.22	0.88	−0.81 (0.36) 0.45	0.023*	0.22	0.90
Body was discovered partly in water	−0.67 (0.56) 0.51	0.23	0.17	1.53	−0.94 (0.59) 0.39	0.113	0.12	1.25
Body was moved	0.69 (0.31) 1.99	0.029*	1.07	3.67	0.79 (0.32) 2.20	0.014*	1.18	4.13
*X* ^2^	68.41***				69.27***			
Nagelkerke *R* ^2^	0.26				0.28			
Overall % predicted	67.20%				67.60%			
ROC‐AUC (con approach)	0.75***	< 0.001	0.69	0.8				

* *p* < 0.05; ** *p* < 0.01; *** *p* < 0.001.

The variable “number of sexual acts” did not pass the Shapiro‐Wilks test (W (323) = 0.88, *p* < 0.001). However, the absolute kurtosis and skewness values remained below two, indicating normality (Skewness = 0.82, Kurtosis = 0.17). Multicollinearity concerns were absent, as all variables had values within the acceptable tolerance threshold (> 0.2) and VIF (< 5.0) (Garson 2016). Furthermore, there was a linear relationship between the variable, “the number of sexual acts,” and the logit of the dependent variable (offender used con‐approach), which was substantiated by a non‐significant Box Tidwell test (*p* = 0.916) (Box and Tidwell 1962). Lastly, the variance of the number of sexual acts committed by con and non‐con approach offenders displayed homogeneity, as the Levene's test was not significant (*p* = 0.604) and equal variances were assumed (*t* [321] = −1.94, *p* = 0.053, Table 2). Consequently, no transformations were applied, indicating the fulfillment of all assumptions for logistic regression.

Among the predictor variables, 8 out of 11 showed statistical significance while controlling for other variables (see Table 3). Female victims had significantly lower odds (OR = 0.18, *p* < 0.001, 95% CI [0.07–0.47]) of being targeted by stranger offenders using a con approach compared to male victims. Similarly, offenders who selected victims engaged in drug and/or alcohol abuse (OR = 2.40, *p* = 0.006, 95% CI [1.29–4.49]) or involved in risky activities such as hitchhiking or the sex trade had over twice the odds of employing a con approach compared to those who did not (OR = 2.91, *p* = 0.022, 95% CI [1.17–7.23]). Whereas victims who were partying before the attack had 69.1% lower odds of being approached by an offender using a ruse (OR = 0.31, *p* = 0.022, 95% CI [0.11–0.84]).

During the crime, offenders who used a con approach had over twice the odds of compelling the victim to perform fellatio compared to those who used other approach tactics (OR = 2.43, *p* = 0.007, 95% CI [1.27–4.65]). On the other hand, those who used a blitz or surprise attack to approach the victim showed a 296% higher likelihood of subjecting the victim to beatings compared to those who used conning tactics (OR = 0.56, *p* = 0.025, 95% CI [0.34–0.93]). In the post‐crime phase, stranger offenders who employed a con approach to engage the victim showed nearly twice the odds of relocating the victim's body (OR = 1.99, *p* = 0.029, 95% CI [1.07–3.67]), and significantly lower odds (56%) of their weapon being recovered by investigators (OR = 0.44, *p* = 0.023, 95% CI [0.22–0.88]) compared to those who used alternative methods of approach. Lastly, the receiver operating characteristics‐area under the curve (ROC) was used to evaluate the performance of the logistic regression model (1 = Yes). The area under the ROC curve was statistically significant at 0.75 (Table 3). According to Rice and Harris (2005), a value of 0.75 puts the discrimination of this model at the lower border of a large effect (0.75, *p* < 0.001, 95% CI [0.69–0.8]) (Table 3).

## 4 Discussion

This study analyzed the behaviors of sexual murderers to identify common patterns of choices made during each phase of the crime based on how the offender approached the victim. The goal was to determine whether it is possible to distinguish between individuals who use conning tactics and those who employ forceful approaches, such as threats, immediate attack, or abduction. Understanding how an offender approached a victim in stranger‐perpetrated homicide is important as it involves engaging with the victim, potentially for extended periods of time. The more information an investigator has, the better, especially since stranger‐perpetrated homicide is among the most challenging to solve (Dietz, Hazelwood, and Warren 1990; Meloy 2000; Salfati and Canter 1999).

Investigators start their investigation by tracing the victim's last known whereabouts, their acquaintances, and anyone who might have had a reason to harm them. As Salfati and Canter (1999) explain, “In the case of stranger homicides, there is little information directly available to investigators other than that available from the crime scene and associated aspects of the crime, such as the details of the victim, the time and the location of the crime” (p. 392). Our results indicate that there are differences in sexual homicide cases that can help predict the methods used by offenders to approach their victims. Early recognition of whether the offender was a stranger to the victim and understanding how that stranger gained access to the victim might not solve the crime, but it can help investigators identify how to prioritize their efforts.

Our findings suggest that individuals who use “cons,” such as pretending to need help, tend to exhibit more deliberate and strategic behaviors, than those using coercive means. For example, they are more inclined to move the victim's body and to have the victim perform oral sex on them. Because these behaviors require additional time and effort, they could be seen as risky and increase the chances of the offender being caught (Martineau and Beauregard 2016). To reduce this risk, conning offenders may choose to target vulnerable individuals or those involved in dangerous activities with strangers, like sex work or hitchhiking, who might go missing for longer periods before anyone notices (Beauregard and Martineau 2014; Quinet 2007, 2011). On the other hand, those who use coercion to contact their victims had behavioral patterns similar to those who murder out of anger or to eliminate witnesses.

### 4.1 Selecting an Approach Style

In sexual homicide cases, the offender typically begins by choosing to approach a victim, even if their initial intention is not to kill them. The way they approach the victim is influenced by various factors, including the victim's perceived vulnerability (physically and/or mentally), and the offender's own level of comfort (MacCulloch et al. 1983). These factors combine to determine the victim approach strategy they will use. Specifically, our study findings indicate that the victim's gender and activities significantly influence the chosen approach.

#### 4.1.1 The Victim's Gender

While most sexual homicide victims are female, our study revealed that the gender of the victim was a significant predictor of the attacker's approach. Those who selected to con a victim had more male victims than those who opted for blitz or surprise attacks. Recent research by Chopin and Beauregard (2021) supports the finding that sexual murderers using a con strategy are likely to target males and show signs of sexual sadism. The difference in gender and approach could be because men tend to resist their aggressors (Beauregard and Proulx 2007; Chopin and Beauregard 2021). When victims resist, it can disrupt the offender's control, making it more difficult for them to complete the crime and increasing the chances of injury, detection, and failure to achieve their objectives (Beauregard, Rossmo, and Proulx 2007; Chopin and Beauregard 2021).

#### 4.1.2 The Victim's Lifestyle and Activities Before the Attack

Victims who appear vulnerable, such as sex trade workers, may have experience dealing with violent individuals. Therefore, they may be willing to fight back, despite being willing to engage with a stranger (see Quinet 2011). This could explain why in this study, one in three of the victims targeted by an offender using a con were classified as vulnerable. On the other hand, blitz or surprise attacks were more commonly used by offenders when the victims were partying. Strangers may appear less approachable if they are with acquaintances, especially to those who are fearful of rejection, embarrassment, or experience performance anxiety (Beauregard and Proulx 2002; MacCulloch et al. 1983; Stefanska et al. 2015). The coercive approach strategies are often associated with offenders classified as “angry” and have built up resentment toward women, who are the most likely victims among those who used blitz or surprise attacks (Beauregard, Rossmo, and Proulx 2007; Beech, Fisher, and Ward 2005; Higgs et al. 2017; Proulx and Beauregard 2009).

### 4.2 The Differences in the Attacks: Con Versus Other Approaches

In studying the behaviors of offenders, it becomes clear that there are significant differences in the ways they carry out their crimes. Two distinct patterns emerge: the use of physical violence and the manipulation of victims to engage in oral sex.

#### 4.2.1 Beating the Victim

In our study, individuals who were found to use blitz and surprise attacks when approaching the victim were significantly more likely to resort to physical beating. The use of physical violence is often employed as a coercive tactic to gain control over a victim, particularly in crimes driven by emotions such as anger or frustration (Beech, Fisher, and Ward 2005; Chopin and Beauregard 2021; Higgs et al. 2017; Proulx and Beauregard 2009; Stefanska et al. 2015). In these instances, violence is strategically used to overcome resistance or avoid detection (Beech, Fisher, and Ward 2005; Chopin and Beauregard 2021; Higgs et al. 2017; Proulx and Beauregard 2009; Stefanska et al. 2015).

#### 4.2.2 Oral Sex

On the other hand, offenders who use a deceptive approach are more likely to persuade their victims to participate in the crime against them. Our study showed that one in four offenders who employed deceptive tactics were able to manipulate their victims into engaging in oral sex, willingly or not, while being less likely to beat the victim. Although the sexual act is not common in cases of sexual homicide, it is considered a sign of sexual sadism (Reale, Beauregard, and Martineau 2017). The finding is significant because it suggests that, similar to sadistic offenders, those who use deception may rely more on psychological manipulation and require less physical violence to achieve their goals, when compared to perpetrators who use different victim targeting strategies (Meloy 2000; Proulx and Beauregard 2009; Reale, Beauregard, and Martineau 2017). However, their control over the situation may weaken over time due to situational factors, resulting in the victim resisting, and might explain why the use of manipulation does not always preclude the use of physical force (Beauregard and Proulx 2007; Reale, Beauregard, and Martineau 2017).

### 4.3 Managing the Evidence After the Crime: Con Versus Other Approaches

The characteristics of offenders with sadistic tendencies includes high levels of organization, strategic planning, prolonged interaction with the victim, and a desire for control (Beauregard and Proulx 2002; Proulx and Beauregard 2009; Ressler, Burgess, and Douglas 1988). As seen in the current study, similar characteristics appear to be linked to an offender's capacity to deceive a victim and to conceal or destroy evidence attempting to avoid detection (Beauregard and Proulx 2002; Beauregard, Rossmo, and Proulx 2007; Meloy 2000). Individuals who use manipulation to target their victims are more likely to find a way to relocate the victim's remains and dispose of any weapons used in the crime, compared to those who immediately attack or abduct the victim. Those who use more forceful tactics more often abandon the victim's body. Their behavior might suggest they did not initially intend to murder the victim (Beauregard and Proulx 2002; Proulx and Beauregard 2009) and therefore, might not have planned what to do with the body (Chai, Beauregard, and Chopin 2020, 707). Instead, these offenders, perhaps panicked, may have weighed the trade‐offs of spending more time at the crime scene and leaving behind evidence (Beauregard and Martineau 2013).

The decisions and behaviors of an offender can impact the time it takes to investigate a case, even though it may eventually be solved. For example, sadistic offenders tend to be more planned (Beauregard and Proulx 2002; Beauregard, Rossmo, and Proulx 2007), but are not necessarily more likely to have their cases go unsolved (Reale, Beauregard, and Martineau 2017). However, their strategies might require investigators to exert more effort to solve the case (Beauregard and Martineau 2014). This may explain why the “con approach” group in our study did not have a higher success rate in avoiding capture than those who used forceful tactics and, were less inclined to clean‐up after the crime (i.e., move the victim's body or successfully dispose of their weapon).

When a victim's body has been moved, it becomes more challenging to locate them and recover forensic evidence due to the time lapse and evidence decay (Beauregard and Martineau 2014; Reale and Beauregard 2018). Additionally, vulnerable victims, who are more likely to be targeted with a ruse, may take longer to be reported missing to the police by loved ones, leading to slowed police response (Beauregard, Proulx, Rossmo, et al. 2007; Martineau and Beauregard 2016). These behaviors complicate solving the crime, and strain police resources, which provides the offender with the time to commit more crimes (Beauregard, Proulx, Rossmo, et al. 2007; Martineau and Beauregard 2016). For example, serial offenders targeting sex workers tend to operate for an average of 9.3 years, almost 2 years longer than those targeting other victims (an average of 7.5 years) (Godwin 2008). Understanding how the offender gained access to the victim may help to minimize delays in the investigation.

### 4.4 Implications

#### 4.4.1 Theoretical Implications

The study findings, at a theoretical level, provide evidence to suggest that individuals who commit sexual murder make rational choices when carrying out their crimes. Specifically, those who employ a “con approach” to access a victim tend to choose targets that are easier to access and lower their risk of being caught (Martineau and Beauregard 2016; Quinet 2011). The versatility of this approach can be advantageous when dealing with a resistant victim, particularly males, as it can help avoid drawing unwanted attention to the situation (Beauregard, Proulx, Rossmo, et al. 2007; Chopin and Beauregard 2021). If initial attempts to access the victim are unsuccessful, the offender may try again. Although it may require more time and effort to access the victim, the payoff lies in the ability to isolate them, affording more time to carry out their sexual fantasies and dispose of evidence (Chopin et al. 2019; Proulx and Beauregard 2009; Salfati et al. 2015). However, the extra time and effort involved in these tasks increase the risk of being detected or having witnesses, but successfully executing them can help in evading or delaying apprehension (Beauregard and Martineau 2013). On the other hand, those who use coercive tactics select victims known to be less likely to be able to resist a physical attack, such as females and victims who have been partying (Beauregard, Proulx, Rossmo, et al. 2007). The benefit to the offender who acts quickly, possibly impulsively, gets immediate access to the victim. These crimes are usually less organized, with perpetrators trying to minimize the time spent committing the crime to reduce the risk of being caught (see Chai, McCuish, and Beauregard 2021; Meloy 2000).

#### 4.4.2 Practical Implications

When creating a suspect list, the police typically check sex offender registries, parolee lists, and other criminal databases (Rossmo 2021). However, not all sexual killers have prior convictions (Greenall and Richardson 2015). For example, Beauregard and Martineau (2013) found that their sample of sexual murderers had an average of 0.4 prior convictions, and almost 80% of the offenders had no prior sexual convictions. Therefore, relying solely on databases may not be sufficient. It could be beneficial for law enforcement to consider alternative avenues including ways to locate additional witnesses, particularly if a perpetrator attempted to access a victim with a ruse but failed.

Understanding the known behaviors of offenders, including their preferred approach, can serve as a predictor of the type of sexual murderer law enforcement may be dealing with. Utilizing investigative tools such as criminal profiles can provide law enforcement with probabilistic insights to guide investigations (Rossmo 2021, 2023). These tools can be particularly helpful in crimes committed by strangers, which often suffer from information overload (Rossmo 2021, 2023). For instance, Bundy was just one suspect among 3500 others (Michaud and Aynesworth 2000). Integrating physical evidence, investigative tools, and accounts from victims and witnesses may not solve the crime, but they can play a vital role (Rossmo 2023). However, using the right tools and identifying potential witnesses who might not realize they hold important information requires investigators to understand the approach strategies and the different types of behaviors exhibited by sexual murderers. Otherwise, law enforcement may be wasting valuable time and their already limited resources.

### 4.5 Limitations and Future Research

While the study provides valuable insights, it is important to recognize that it is exploratory and sets the stage for future research. The findings shed light on the strategic behaviors of offenders who use the “con” approach, as well as the less organized efforts of other strategies, such as blitz and surprise attacks. However, despite their calculated approach, these individuals were not more successful in evading capture than their counterparts. Therefore, the successful execution of these tactics remains unclear, given that most cases were solved.

While the study focused on single‐offense sexual homicides, it is important to acknowledge the possibility that some offenders may have committed additional murders for which they remain unconnected. The complexities of identifying and understanding patterns in serial offenses have been the focus of previous research (e.g. Salfati et al. 2015); however, nuances also exist in the dynamics of single‐offense sexual homicides. Future research could benefit from exploring these nuances further, particularly by examining combined approach methods. For example, an offender might deceive the victim by pretending to be someone else to gain access to their home (a con) and then immediately attack the victim upon entry.

Additionally, incorporating contextual and situational data, such as the amount of time the perpetrator spent with the victim, could help narrow down the evidence collection timeframe or when the crime may have occurred. Understanding the duration of the interaction could make it easier to determine if specific actions, such as oral sex, occurred simply because the offender had more time to manipulate the victim, or if the interaction was initially consensual and then turned violent. The amount of time is particularly important, since previous research has indicated that sadistic offenders who frequently use deceitful tactics to approach their victims tend to spend more time with them (see Proulx and Beauregard 2009). Law enforcement could benefit from testing these findings to refine investigative strategies and enhance effectiveness.

## Author Contributions

**Zena Rossouw:** responsible for the conception of the study, interpretation of data, and writing. **Eric Beauregard:** supervision, study conception, design, data management, review, and feedback. **Julien Chopin:** data curation, data management, review and feedback for the study.

## Ethics Statement

This study was reviewed and approved by the Simon Fraser University Office of Research Ethics, and designated as minimal risk (Protocol #[30002127], December 12, 2023).

## Conflicts of Interest

The authors declare no conflicts of interest.
